# Among the Board of Examiners

**Published:** 1902-11

**Authors:** 


### AMONG THE BOARDS OF EXAMINERS.

President William Herbert Lowe has called a two-days' session of the New Jersey State Board of Veterinary Medical Examiners, to be held at the State House, Trenton, N. J., January 9 and 10, 1903, for the purpose of examining candidates for license to practice veterinary medicine, surgery, and dentistry in that State.

The Pennsylvania State Board of Veterinary Medical Examiners will hold their semi-annual examination in Philadelphia, Pa., the third Monday and Tuesday of December.

---

Veterinary Surgeon Desmond, of Adelaide, South Australia, continues to do excellent work for the people in that country. His investigations of all reported outbreaks of contagious and infectious diseases among animals, and subsequent valuable reports to the national and local boards of health, are complete, interesting, and educational, and destined to raise veterinary science in public estimation.

